# The relationship between dietary phosphorus and peripheral neuropathy in the general population of the United States: A preliminary research

**DOI:** 10.1371/journal.pone.0299566

**Published:** 2024-03-15

**Authors:** Chunli Wu, Zhe Wu, Yanling Chen, Huirong Xu, Kejian Li

**Affiliations:** 1 The Traditional Chinese Medicine College, Shandong University of Traditional Chinese Medicine, Jinan, China; 2 Department of Endocrinology, Jinan City Hospital of Integrated Traditional Chinese and Western Medicine, Jinan, China; 3 The First Clinical College, Shandong University of Traditional Chinese Medicine, Jinan, China; University of Life Sciences in Lublin, POLAND

## Abstract

**Background:**

Dietary phosphorus intake may serve as a potential predictor for peripheral neuropathy (PN). While past research has predominantly focused on the relationship between dietary phosphorus and bone health, relatively little is known about its role in the nervous system, particularly its association with PN.

**Methods:**

A cross-sectional study was conducted using data from NHANES 1999–2004. Participants were categorized into different dietary phosphorus intake groups, and the relationship between dietary phosphorus and PN was explored using multifactorial logistic regression, restricted cubic splines (RCS) analysis, and threshold effect analysis based on dietary intake.

**Results:**

The final study included 7726 participants, with 1378 diagnosed with PN and 6348 without. The study revealed a U-shaped non-linear relationship between dietary calcium and magnesium intake levels and PN, indicating that both excessive and insufficient dietary phosphorus intake may increase the risk of PN. Specifically, the incidence rates in the first quintile (1.433, 95% CI: 1.080–1.901), the fourth quintile (1.284, 95% CI: 1.000–1.648), and the fifth quintile (1.533, 95% CI: 1.155–2.035) significantly higher than the second quintile, with an overall trend showing a decrease followed by an increase in incidence rates. The results of RCS and threshold effect analysis indicate that when dietary phosphorus intake is below 939.44mg, the risk of PN decreases with increasing dietary phosphorus intake. On the contrary, when dietary phosphorus intake exceeds 939.44mg, the risk of PN increases with increasing dietary phosphorus intake.

**Conclusion:**

This study reveals a U-shaped correlation between dietary phosphorus intake and PN. Future research should further elucidate the molecular mechanisms underlying this association, providing guidance for more scientifically informed dietary adjustments to prevent the occurrence of PN.

## Background

In contemporary society, the connection between diet and health is receiving increasing attention, and people are becoming more concerned about the impact of the intake of various nutrients in the diet on different systems of the body. Phosphorus is a crucial element for maintaining bone structure, cellular metabolism, and energy conversion [1, 2]. With the development of society and the improvement of living standards, there has been a noticeable change in the dietary structure of American residents. Traditional dietary habits have been replaced by more processed foods rich in phosphates. This has led to a significant increase in the daily intake of phosphorus compared to the past [3]. Previous research has primarily focused on the relationship between dietary phosphorus and bone health, emphasizing the role of phosphorus in maintaining bone density and skeletal structure [4, 5]. However, there is relatively limited understanding of its role in the nervous system, especially its impact on the peripheral nervous system. In recent years, research on the relationship between dietary phosphorus and nervous system health has attracted increasing attention.

Peripheral neuropathy (PN) is a category of diseases that affect the peripheral nerves, clinically manifesting as numbness, sensory abnormalities, muscle weakness, motor disturbances, and chronic ulcers [6–9]. These diseases not only directly impact the physical functions of patients but also have profound negative effects on their quality of life. Therefore, investigating the pathogenesis of PN, especially its relationship with dietary phosphorus, holds positive significance for improving the quality of life for patients.

This study aims to utilize data from NHANES 1999–2004 to delve into the relationship between dietary phosphorus and PN in the general population of the United States, with the goal of reducing the risk of PN.

## Method

### Data sources

NHANES is a nationwide survey organized by the National Center for Health Statistics (NCHS) in the United States, aimed at assessing the health and nutritional status of Americans. The data we analyzed comes from the collection of NHANES cycles from 1999 to 2004. The research protocol of Nhanes 1999–2004 was approved by NCHS. According to local laws, this second analysis does not require further approval from the institutional review committee.

### Peripheral neuropathy

The evaluation of PN was conducted by health technicians using standard monofilament (Semmes Weinstein nylon, size 5.07) to apply pressure to three areas of each foot to test the subject’s foot sensation. The presence of PN is defined as having at least one non sensory area on both feet.

### Dietary phosphorus intake

The information about the phosphorus intake of participants was estimated using the 24-hour dietary recall method. Calculate dietary phosphorus intake based on all food and beverages consumed by participants in the past 24 hours. The NHANES Dietary Access Program Manual provides a detailed explanation of this process.

### Covariate

The covariates considered in the analysis include age, gender, race (white, black, Mexican American or other races), smoking, alcohol consumption, body mass index (BMI), and whether or not they have cardiovascular disease (CVD), hypertension, diabetes, and chronic kidney disease (CKD). CVD is determined through a questionnaire. Hypertension is defined as an average systolic blood pressure of ≥ 140 millimeters of mercury and an average diastolic blood pressure of ≥ 90 millimeters of mercury, diagnosed by a doctor, or using antihypertensive drugs. Diabetes is defined as random blood glucose ≥ 11.1 mmol/L or fasting blood glucose ≥ 7 mmol/L or glycosylated hemoglobin>6.5% or two-hour OGTT blood glucose ≥ 11.1 mmol/L, or diagnosed by a doctor, or using hypoglycemic drugs. CKD is defined as glomerular filtration rate < 60ml/min/1.73m^2^.

### Statistical analysis

Statistical analysis was conducted using R studio (4.2.1) to analyze the data, with weighting based on dietary factors. Continuous variables are represented as mean (standard error), categorical variables are represented as mean (percentage). Based on the dietary intake of phosphorus, we divide the population into five percentiles. The critical values for the five quantiles are Q1 ≤ 720.13mg, 720.13mg < Q2 ≤ 978mg, 978mg < Q3 ≤ 1245.23mg, 1245.23mg < Q4 ≤ 1613.77mg, Q5 > 1613.77mg. We used a multiple logistic regression model to analyze the total phosphorus intake in the diet and the relationship between each quantile and PN. To illustrate the dose-response relationship between dietary phosphorus intake and PN, we used restricted cubic splines (RCS).

## Results

### Baseline information

A total of 9145 subjects participated in the assessment of PN. We excluded subjects who could not provide peripheral nerve information (n = 1243) and those lacking relevant dietary data (n = 176). Ultimately, the study included 7726 subjects, among whom 1378 had PN, and 6348 did not. Table 1 displays the basic characteristics of the participants. Participants were grouped based on the presence or absence of PN. Significant differences were observed between the two groups in terms of age, gender, BMI, smoking, alcohol consumption, hypertension, diabetes, cardiovascular diseases, and CKD prevalence. However, there was no significant difference in dietary phosphorus intake between the two groups. Results from the five quintile groups of dietary phosphorus intake (Table 2) showed a lower incidence of PN at the second quintile, with an overall trend of decreasing and then increasing incidence, suggesting a potential non-linear relationship between dietary phosphorus intake and PN.

**Table 1:** Population characteristics stratified by PN.

Variable	Total	Non-PN	PN	*P*-value
**Age years**	57.15(0.25)	56.03(0.24)	64.10(0.52)	< 0.0001
**Sex**				< 0.0001
Female	3882(50.25)	3363(54.80)	519(38.77)	
Male	3844(49.75)	2985(45.20)	859(61.23)	
**Race**				0.45
White	4262(55.16)	3506(78.32)	756(76.89)	
Black	1384(17.91)	1127 (9.26)	257(10.81)	
Mexican American	1577(20.41)	1286(4.40)	291(4.55)	
Other Race	503(6.51)	429(8.01)	74(7.74)	
**BMI kg/m** ^**2**^	28.49(0.12)	28.34(0.13)	29.47(0.23)	< 0.0001
**Dietary phosphorus intake mg**	1261.17(11.45)	1257.82(12.65)	1281.96(26.23)	0.41
**Smoke**				0.02
Former	2654(34.4)	2109(33.04)	545(39.17)	
Never	3592(46.55)	2971(46.55)	621(42.83)	
Now	1470(19.05)	1259(20.41)	211(18.00)	
**Alcohol consumption**				< 0.0001
Formor	1937(25.52)	1507(21.37)	430(28.24)	
Never	1120(14.75)	878(12.45)	242(17.70)	
Now	4534(59.73)	3859(66.18)	675(54.06)	
**Hypertension**				< 0.0001
No	3411(44.17)	2938(51.87)	473(39.03)	
Yes	4312(55.83)	3408(48.13)	904(60.97)	
**Diabetes**				< 0.0001
No	6294(81.54)	5334(87.93)	960(71.55)	
Yes	1425(18.46)	1008(12.07)	417(28.45)	
**CKD**				< 0.0001
No	6337(85.98)	5340(91.16)	997(79.20)	
Yes	1033(14.02)	714 (8.84)	319(20.80)	
**CVD**				< 0.0001
No	6422(83.13)	5423(87.58)	999(74.09)	
Yes	1303(16.87)	924(12.42)	379(25.91)	

**Table 2:** Population characteristics stratified by dietary phosphorus intake.

Variable	Q1	Q2	Q3	Q4	Q5	*P*-value
**Age years**	59.47(0.48)	59.07(0.45)	57.91(0.39)	56.62(0.41)	53.81(0.40)	< 0.0001
**Sex**						< 0.0001
Female	1055(74.23)	964(66.58)	814(54.98)	633(45.05)	416(30.63)	
Male	491(25.77)	582(33.42)	730(45.02)	912(54.95)	1129(69.37)	
**Race**						< 0.0001
White	656(65.37)	817(76.24)	871(78.14)	940(82.15)	978(85.25)	
Black	439(17.21)	321(11.18)	254 (9.09)	193 (6.75)	177 (5.27)	
Mexican American	325(4.81)	303(4.42)	305(4.08)	330(4.77)	314(4.14)	
Other Race	126(12.60)	105 (8.16)	114 (8.69)	82 (6.33)	76 (5.34)	
**BMI kg/m** ^**2**^	28.31(0.22)	28.49(0.20)	28.21(0.23)	28.84(0.22)	28.58(0.29)	0.27
**Smoke**						< 0.001
Former	457(26.97)	490(30.43)	548(34.45)	577(38.76)	582(36.84)	
Never	770(48.92)	772(49.47)	704(45.87)	676(42.15)	670(44.81)	
Now	316(24.11)	282(20.09)	291(19.68)	289(19.09)	292(18.35)	
**Alcohol consumption**						< 0.0001
Formor	439(25.53)	412(24.40)	371(21.52)	360(19.75)	355(21.34)	
Never	319(20.15)	289(17.95)	211(11.59)	165 (9.85)	136 (8.67)	
Now	755(54.32)	819(57.66)	933(66.89)	1002(70.40)	1025(69.99)	
**Hypertension**						< 0.0001
No	570(42.77)	606(47.00)	716(52.01)	682(49.32)	837(56.86)	
Yes	975(57.23)	940(53.00)	828(47.99)	861(50.68)	708(43.14)	
**Diabetes**						0.34
No	1233(84.00)	1236(84.76)	1259(85.62)	1274(86.64)	1292(86.71)	
Yes	311(16.00)	309(15.24)	284(14.38)	270(13.36)	251(13.29)	
**CKD**						< 0.0001
No	1166(82.74)	1230(87.83)	1263(88.44)	1310(91.87)	1368(94.35)	
Yes	284(17.26)	249(12.17)	216(11.56)	168 (8.13)	116 (5.65)	
**CVD**						< 0.0001
No	1229(81.39)	1267(84.92)	1290(85.23)	1298(86.63)	1338(89.01)	
Yes	317(18.61)	279(15.08)	254(14.77)	247(13.37)	206(10.99)	
**PN**						0.11
No	1233(84.33)	1296(88.24)	1274(87.00)	1266(85.70)	1279(85.38)	
Yes	313(15.67)	250(11.76)	270(13.00)	279(14.30)	266(14.62)	

### Multivariate logistic regression

To further explore the relationship between dietary phosphorus intake and PN, we conducted multivariate logistic regression. Both unadjusted and fully adjusted models showed no significant correlation between dietary phosphorus intake and PN (Table 3). When dietary phosphorus intake was divided into quintiles without adjusting for covariates, the incidence rates of PN in the first quintile (1.395, 95% CI: 1.073–1.812) and the fifth quintile (1.286, 95% CI: 1.017–1.625) were significantly higher compared to the second quintile. In the fully adjusted model, the incidence rates in the first quintile (1.433, 95% CI: 1.080–1.901), the fourth quintile (1.284, 95% CI: 1.000–1.648), and the fifth quintile (1.533, 95% CI: 1.155–2.035) remained significantly higher than the second quintile. This demonstrates a non-linear relationship between dietary phosphorus intake and PN.

**Table 3:** Relationship between dietary phosphorus intake and peripheral neuropathy.

Result	Model 1		Model 2		Model 3	
	OR(95%CI)	*P*-value	OR(95%CI)	*P*-value	OR(95%CI)	*P*-value
Dietary phosphorus intake	1.000(1.000,1.000)	0.410	1.000(1.000,1.000)	0.116	1.000(1.000,1.000)	0.097
Q2	ref	ref	ref	ref	ref	ref
Q1	1.395(1.073,1.812)	0.014	1.433(1.087,1.888)	0.012	1.433(1.080,1.901)	0.015
Q3	1.121(0.866,1.452)	0.376	1.133(0.852,1.507)	0.380	1.185(0.865,1.624)	0.279
Q4	1.253(0.988,1.588)	0.062	1.275(0.995,1.632)	0.054	1.284(1.000,1.648)	0.050
Q5	1.286(1.017,1.625)	0.036	1.451(1.104,1.908)	0.009	1.533(1.155,2.035)	0.005

Adjusted variables: Model 1: unadjusted. Model 2: age, sex, race. Model 3: all.

OR, odds ratio; CI, confidence interval.

### RCS

**Fig 1:**
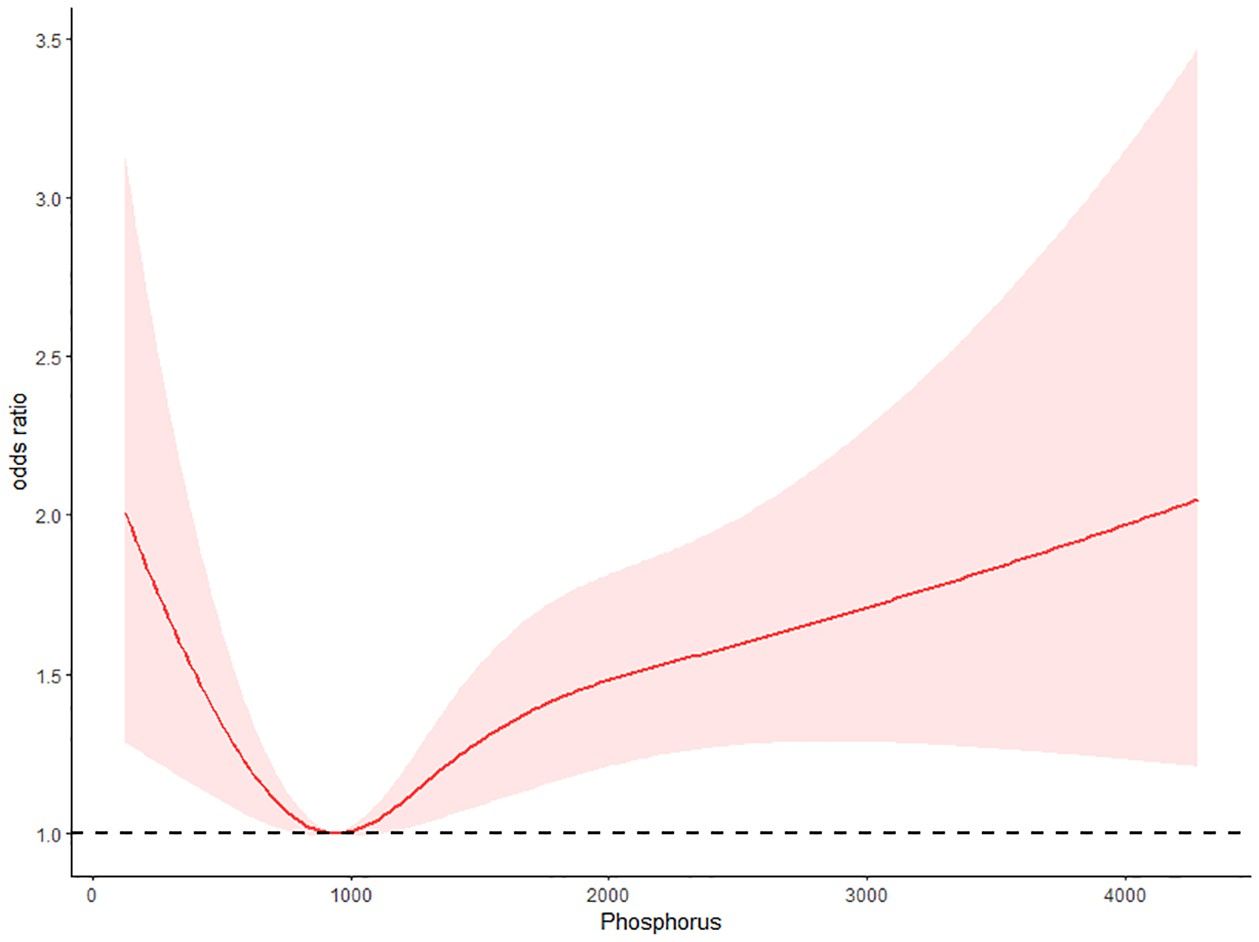
The dose-response relationship between dietary phosphorus intake and PN.

To validate the dose-response relationship between dietary phosphorus intake and PN, we conducted RCS analysis. The results showed that as dietary phosphorus intake increased, the incidence of PN initially decreased and then increased (*P* for nonlinear < 0.001). When dietary phosphorus intake was less than 939.44mg, the risk of PN decreased with increasing dietary phosphorus intake, while when dietary phosphorus intake was greater than 939.44mg, the risk of PN increased with increasing dietary phosphorus intake (Fig 1).

### Threshold effect analysis

Threshold effect analysis revealed a significant reduction in the risk of PN with increasing dietary phosphorus intake when it was less than 939.44mg (*P* = 0.005). Conversely, when dietary phosphorus intake exceeded 939.44mg, the risk of PN significantly increased with increasing dietary phosphorus intake (*P* = 0.025). (Table 4).

**Table 4:** Threshold effect analysis.

Character	OR (95% CI)	*P*-value
Dietary phosphorus intake		
≧ 939.44mg	1.000(1.000,1.000)	0.025
< 939.44mg	0.999(0.998,1.000)	0.005

OR, odds ratio; CI, confidence interval.

## Discussion

In this cross-sectional study, we aimed to investigate the relationship between dietary phosphorus intake and PN in the general population of the United States. Our study results indicate a U-shaped non-linear relationship between dietary intake levels of phosphorus and PN, suggesting that both excessive and insufficient phosphorus intake may increase the incidence of PN. To our knowledge, this is the first study exploring the relationship between dietary phosphorus and PN in the general population.

Our study differs from previous research on dietary phosphorus and diseases. Past studies primarily focused on the risks associated with excessive dietary phosphorus, while our research emphasizes that insufficient dietary phosphorus intake may also have negative effects on the nervous system [10–13]. This finding provides a basis for establishing more comprehensive dietary guidelines, cautioning individuals not only about the risks of excessive phosphorus intake but also the importance of ensuring sufficient dietary phosphorus to maintain neurological health.

Excessive dietary phosphorus intake has been linked to various health issues, including osteoporosis and cardiovascular diseases [14]. We speculate that elevated serum phosphorus due to excessive dietary intake may trigger inflammatory reactions and oxidative stress, negatively impacting the peripheral nervous system [15–17]. Additionally, phosphorus may influence the occurrence of PN by regulating the function of neuronal cell membranes and affecting neural signal transmission. Conversely, insufficient dietary phosphorus intake may also have negative effects on the nervous system, as phosphorus plays a crucial role in maintaining cell membrane structure, ATP synthesis, and other processes vital for normal neuronal function [18].

Our study reveals that the incidence of PN appears to be much higher than previously recognized [19–21]. PN is commonly associated with diabetes, but our research indicates that the majority of PN cases are in non-diabetic individuals. Among diabetic patients, only 13.29% have PN. This suggests that neuropathy caused by other factors may be significantly underestimated or overlooked.

We must acknowledge the limitations of this study. One major limitation is its cross-sectional nature, which prevents the determination of the mechanisms underlying the correlation between dietary phosphorus intake and PN. Additionally, using 24-hour dietary recall to determine phosphorus intake may introduce some error.

## Conclusion

Our study reveals a U-shaped correlation between dietary phosphorus intake and PN. This finding provides new insights for a deeper understanding of the relationship between dietary phosphorus and PN. Future research can further elucidate the molecular mechanisms of this association and guide more scientifically informed dietary adjustments to prevent the occurrence of PN in clinical practice.

## Supporting information

S1 Data (XLSX)
